# Personal views of aging and quality of life in midlife and older age: the role of cognitive reserve

**DOI:** 10.3389/fpsyg.2026.1778263

**Published:** 2026-03-18

**Authors:** Elena Carbone, Enrico Sella, Paolo Ghisletta, Erika Borella

**Affiliations:** 1 Department of General Psychology, University of Padova, Padua, Italy; 2 Faculty of Psychology and Educational Sciences, University of Geneva, Geneva, Switzerland

**Keywords:** felt age, attitudes toward own aging, awareness of age-related change, cognitive reserve proxies, quality of life

## Abstract

**Introduction:**

This study examined the relationships between different personal views of aging (VoA) dimensions, quality of life (QoL), and cognitive reserve (CR) proxies across the adult life span. In particular, we explored the role of CR proxies as a pathway mediating the VoA–QoL associations.

**Methods:**

A sample of 552 participants (50–84 years) reported their felt age and completed the Attitudes Toward Own Aging scale (ATOA) and Awareness of Age-Related Change questionnaire (AARC) as measures of personal VoA; they also filled in the World Health Organization Quality of Life questionnaire. Participants also completed the Current and Retrospective Cognitive Reserve survey (2CR), assessing various CR proxies both currently (CR-current) and retrospectively (CR-retrospective).

**Results:**

Path analyses showed direct effects of VoA, in particular ATOA, AARC-Gains, and AARC-Losses (but not felt age), on QoL. Positive ATOA were related to better QoL through greater AARC-Gains, whereas negative ATOA and an older felt age predicted poorer QoL through greater AARC-Losses. Direct effects of personal VoA on CR also emerged, in particular ATOA, AARC-Gains on CR-current, and ATOA, AARC-Losses and felt age on CR-retrospective. Both CR-current and CR-retrospective mediated the effect of ATOA on QoL, whereas only CR-current mediated the effect of AARC-Gains on QoL. Finally, chronological age related to CR-retrospective and explained QoL only indirectly through ATOA and AARC.

**Conclusion:**

Our results, beyond confirming the relevance of personal VoA for QoL, suggest CR proxies as a possible behavioural pathway linking VoA to QoL. However, VoA, CR and QoL relationships depend on the VoA dimensions, and current or retrospective CR proxies considered. Ensuring quality of life, and thus successful/healthy aging, across adult lifespan should also account for views of aging and cognitive reserve proxies.

## 1 Introduction

Personal views of aging (VoA)—that is, individuals’ perceptions, attitudes, and expectations of one’s own aging process related to their lifelong experiences—(Kornadt et al., 2020; Shrira et al., 2022) are a well-established influence of various physical, psychological, quality of life (QoL) and longevity outcomes across the adult lifespan—in midlife and older age—(e.g., Westerhof et al., 2023; Sabatini et al., 2020). They encompass a variety of related yet distinct constructs, namely felt age, attitudes toward own aging (ATOA), and awareness of age-related change (AARC) (see Sella et al., 2025b). Felt age, or the discrepancy between an individual’s perceived and actual chronological age, and ATOA, or individuals’ behavioural, cognitive, and affective evaluations and expectations about their experience of growing older and being older adults, represent global evaluations of one’s own aging process (Diehl et al., 2014). The more recent AARC construct, instead, depicts behaviour-specific VoA, that is self-reflections and conscious awareness of aging-related changes in terms of gains (AARC-Gains) and losses (AARC-Losses) grounded in directly lived, lifelong daily-life experiences across various domains of functioning (Brothers et al., 2017; Diehl et al., 2014). Both global and behaviour-specific personal VoA constructs have been shown to be relevant for a range of outcomes closely related to QoL: feeling younger than one’s chronological age, or holding positive ATOA have been found to be positively associated with various health-related indicators, such as better physical health, reduced depressive symptoms, and better cognitive functioning (e.g., Debreczeni and Bailey, 2021; Fernández-Ballbé et al., 2023; Tully-Wilson et al., 2021). Similarly, greater AARC-Gains and AARC-Losses has been shown to be positively and negatively linked, respectively, to health-related outcomes in terms of physical and psychological functioning (Sabatini et al., 2020), and also, though modestly, to cognitive functioning (Carbone et al., 2025a; Fernández-Ballbé et al., 2023). Interestingly, when accounting for the interrelationships among various personal VoA dimensions, behaviour-specific personal VoA, i.e., AARC, likely mediate the link between global personal VoA dimensions (i.e., felt age and ATOA) and physical or psychological/mental health outcomes (see Brothers et al., 2017; Carbone et al., 2025a).

Personal VoA are, thus, clearly associated to physical and psychological health outcomes closely related to QoL. There is now also increasing evidence of meaningful associations between VoA and the broad concept of QoL, that is the multidimensional subjective perception that individuals have of their position in life in various physical, mental, as well as social domains of functioning according to their goals, expectations, standards, and concerns in the context of the culture system in which they live (Skevington et al., 2004). Most of the extant studies have shown that positive attitudes toward aging are associated with better overall QoL, whereas negative attitudes toward aging are linked to poor overall QoL (see Velaithan et al., 2024 for a review). The only one study, which considered different personal VoA -felt age and AARC- in relation to overall QoL among middle-aged and older individuals, found weak felt age–QoL associations, but holding greater AARC-Gains and lower AARC-Losses were shown to be associated with better overall QoL (Sella et al., 2025a). Nonetheless, the interplay among various global and behaviour-specific personal VoA dimensions and their combined effects on overall QoL requires further examination.

Apart from establishing their synergic impact on QoL in adulthood and older age, understanding the pathways linking personal VoA to QoL represents another underexplored yet emerging issue (Westerhof et al., 2023; Wurm et al., 2017). According to the extant VoA theoretical framework (Shrira et al., 2022; Sabatini et al., 2025), personal VoA would operate, among others, through psychological (see Sella et al., 2025a) and behavioural pathways capturing how VoA contribute to health-related and QoL outcomes over time. In particular, behavioural pathways reflect how VoA would relate to the engagement in all those health-enhancing and adaptive behaviours, such as intellectually stimulating, social, and physical activity (Sabatini et al., 2025; Westerhof et al., 2023). If adopted and cultivated, these latter socio-behavioural indicators depicting lifelong experiences in various contexts (e.g., educational, occupational, free time) are known to contribute building up cognitive reserve (CR). CR is defined as the adaptability of cognitive processes to age-related changes or pathology-related insult due to aging process (Stern et al., 2020). CR not only account for variability in aging-related cognitive changes or neurodegenerative disorders and is known to support cognitive performance (Borella et al., 2023; Stern, 2009, 2019, 2020; Panico et al., 2023), but also plays a protective role by promoting better mood functioning (Borella et al., 2023), psychological wellbeing and life satisfaction (Feraco et al., 2024; Gümüş Demir and Çebi, 2025). Importantly, like VoA, CR represents a dynamic, life-course construct shaped by multiple experiences accumulated across the lifespan (Borella et al., 2023; Ihle et al., 2021; Stern et al., 2020). Therefore, CR is commonly indexed through a variety of socio-behavioural proxies that include socio-economic indicators (educational attainment, occupational complexity, financial wellbeing), leisure cognitive, physical, and social/familiar engagement and, though more rarely, also spiritual/religious activity (Borella et al., 2023), accrued at different life stages such that their contribution to CR building varies across life periods (e.g., Borella et al., 2023; Litkouhi et al., 2024; Stern et al., 2019). Later-life CR proxies (occupational attainment, current family engagement, leisure, social and spiritual engagement), for instance, have been suggested to play a more “protective role” than peak/prior ones (educational attainment, youthful leisure, social and spiritual engagement) in promoting better QoL-related psychological outcomes (Borella et al., 2023; Feraco et al., 2024).

So far, a few studies have attempted to empirically examine the direct associations between personal VoA and some specific CR proxy measures in midlife and older age (see Shoushtari-Moghaddam et al., 2022 for a review). There is some evidence pointing to a youthful felt age, positive ATOA, greater AARC-Gains and lower AARC-Losses being modestly related to early-life CR proxies such as higher educational, or later-life CR proxies like occupational attainment, or financial wellbeing (e.g., see Sabatini et al., 2023a; English et al., 2019; Barrett, 2003). Most previous studies have also shown that more positive ATOA are associated with later-life/current CR proxies related to greater engagement in either leisure cognitive, physical, or social activity (e.g., Beyer et al., 2015; Bu et al., 2024; Huo and Kim, 2022; Schwartz et al., 2021; Sabatini et al., 2023b). Moreover, a youthful felt age was found to be associated with CR proxies related to greater physical activity (Wienert et al., 2017) and participation in social activities (Vaartio-Rajalin et al., 2024). Gain- and loss-oriented self-perceptions of aging assessments showed nuanced associations with CR proxies such as leisure engagement in a variety of enjoyable activities (Windsor et al., 2022), or engagement in social (Sabatini et al., 2023b), leisure and cognitively stimulating activities (Robertson and Kenny, 2016). Of note, few extant studies have also examined the extent to which the associations between personal VoA and indicators closely related to QoL would be mediated by socio-behavioural proxies of CR. These latter have shown that middle-aged and older adults holding more positive personal VoA (e.g., ATOA in Beyer et al., 2015; Expectations Regarding Aging in Kim, 2009; Li et al., 2013), have a higher CR, that is more likely engage in specific socio-behavioural experiences contributing to build up CR—physical activity (e.g., Beyer et al., 2015; Li et al., 2013) or engagement in physical activity, diet, social relationships, and spiritual growth (Kim, 2009), which in turn accounted for better mental, physical, or functional health. More recently, a few studies have shown that higher CR, though indexed through a global composite score integrating proxies spanning both early-life (e.g., educational attainment) and later-life experiences (e.g., occupational complexity and leisure activities), is positively associated also with better overall QoL (Gattuso et al., 2024; Lara et al., 2017; see also Marzo et al., 2023 for a review). However, the extent to which life-stage dependent CR proxies relate differently to overall QoL and may represent a pathway linking personal VoA to overall QoL remains to be fully examined.

Overall, the impact that personal VoA could exert on QoL is clear, and evidence suggests a behavioural pathway linking personal VoA to QoL *via* enriching socio-behavioural experiences that act as proxies of CR. However, further investigation is needed to clarify the extent to which early- and later-life CR proxies relate to both personal VoA and overall QoL, and to elucidate whether and how different personal VoA may impact QoL by drawing on CR-related resources built upon socio-behavioural experiences accumulated at different lifetime points. Therefore, the interplay between various global *vs* behaviour-specific personal VoA dimensions together in explaining overall QoL, and the potential role of life-stage dependent CR proxies in mediating their associations requires further exploration within a more comprehensive, integrative modelling approach as advocated by recent theoretical VoA works (e.g., Kornadt et al., 2020; Shrira et al., 2022; Westerhof et al., 2023; Wurm et al., 2017).

To fill in these gaps, the present cross-sectional study aimed at concurrently investigating the relationships among various personal VoA dimensions, life-stage dependent CR proxies and overall QoL in community-dwelling middle-aged and older adults. The main aim was to examine the associations between overall QoL and both personal VoA and life-stage dependent CR proxies, and the role of life-stage dependent CR proxies in mediating the VoA-QoL associations. The associations between global, i.e., felt age, ATOA, and behaviour-specific, i.e., AARC, personal VoA dimensions and overall QoL, and the mediating role of AARC in the associations between global personal VoA (felt age, ATOA) and QoL were examined as a corollary aim. Towards these aims, well-proven measures to assess personal VoA (see Sella et al., 2025a for a review), i.e., a single-item question for felt age, the Attitudes Towards Own Aging scale (ATOA; Lawton, 1975) and the Awareness of Age-Related Change questionnaire (AARC; Carbone et al., 2024, 2025b), and QoL, i.e., the World Health Organization Quality of Life-Bref assessment (Skevington et al., 2004), were employed. To account for the multidimensional and dynamic nature of CR, the Current and Retrospective Cognitive Reserve survey (2CR, Borella et al., 2023) was adopted, since it allows to capture individuals’ enriching socio-behavioural life experiences known to build up CR not only as they have been accumulated in early life (retrospectively assessed), but also as they currently manifest (at the time of assessment). Additionally, we examined the direct effects of chronological age on overall QoL, as well as any indirect effects through personal VoA dimensions and current or retrospective CR proxies.

According to previous evidence (e.g., Brothers et al., 2017; Carbone et al., 2025a), we could expect significant yet small-to-medium associations among the different personal VoA dimensions considered. In line with previous studies, we expected: (i) positive personal VoA, particularly ATOA and AARC, to be directly associated with better QoL (see Velaithan et al., 2024; Sella et al., 2025a), and (ii) AARC to mediate the link between global VoA and QoL (see Brothers et al., 2017; Carbone et al., 2025a). In particular, a youthful felt age and positive ATOA were expected to be associated with better overall QoL through aging-related daily-life experiences in terms of greater AARC-Gains, whereas an older felt age and negative ATOA could be associated with poorer overall QoL through aging-related daily-life experiences of greater AARC-Losses (Brothers et al., 2017; Carbone et al., 2025a). Then, we hypothesized: (iii) positive associations between personal VoA and CR proxies, in line with extant evidence (e.g., Beyer et al., 2015; Bu et al., 2024; Robertson and Kenny, 2016; Shoushtari-Moghaddam et al., 2022; Wienert et al., 2017); (iv) positive associations between CR and QoL, according to previous studies (Barrett, 2003; Lara et al., 2017; Gattuso et al., 2024; Sabatini et al., 2023a); and (v) that, considering current VoA theoretical framework (Sabatini et al., 2025; Westerhof et al., 2023) and previous findings (Kim, 2009; Beyer et al., 2015; Li et al., 2013), positive personal VoA could relate to a greater reliance on both current and retrospectively accumulated CR resources through engagement in socio-behavioural experiences at different points of one’s lifetime and, in turn, better QoL, whereby reflecting a behavioural pathway linking VoA to QoL. We explored whether various pathways linking different personal VoA to QoL *via* current *vs* retrospective CR proxies may emerge.

Finally, chronological age *per se* was not expected to directly impact overall QoL, according to previous evidence (see Sella et al., 2025a). We assumed an older chronological age to indirectly impact QoL, through its direct and negative influence on evaluations of one’s own aging process due to increased physical, psychological, and social challenges (e.g., Brothers et al., 2017). An older chronological age was, then, expected to be associated with lower CR-retrospective, but not CR-current, as previously found (see Borella et al., 2023). Finally, we explored the potential interplay between chronological age, current *vs* retrospective CR proxies, and overall QoL.

## 2 Method

### 2.1 Participants

This cross-sectional study involved 552 typically-aging community-dwelling middle aged and older adults (age range: 50–84 years; 64% female), who volunteered for the study. All participants were native Italian speakers recruited through informal interpersonal dissemination (word-of-mouth), including personal and professional networks and acquaintances.

Eligibility criteria included: (i) a good physical and mental health status, assessed with a semi-structured interview (De Beni et al., 2008) asking participants to report, for example, whether they had a history of psychiatric or neurological disorders or other diseases causing cognitive impairments, visual, auditory, and/or motor impairments, serious health issues and/or use of medication; (ii) a Mini-Mental State Examination (MMSE; Frisoni et al., 1993) score ≥ 27, i.e., no signs of mild/major neurocognitive disorders -adopting a more conservative cut-off than the standard ≥ 26 (e.g., Foderaro et al., 2022) to improve the discriminative properties of our inclusion criteria for selecting participants without cognitive impairment-; and (iii) a Geriatric Depression scale (Yesavage et al., 1982; Sheikh and Yesavage 1986) score ≤ 5, i.e., no signs of major depressive symptoms.

The study was approved by the local Ethics Committee and conducted in accordance with the Declaration of Helsinki. All participants were informed about the aims of the study and provided written informed consent prior to participation.

Table 1 shows the descriptive statistics of participants’ socio-demographic characteristics and the measures of interest.

**Table 1:** Descriptive statistics of participants’ socio-demographic characteristics, screening measures and the measures of interest.

	*M*	SD
Socio-demographic characteristics		
Chronological age	63.801	8.196
Education (years)	11.596	4.021
Gender, *n* females (%)	353 (64%)	–
Retirement (yes, %)	242 (44%)	–
Screening measures		
MMSE	29.35	1.01
GDS	1.51	1.43
Views of aging		
Felt age	−0.138	0.146
ATOA	12.413	1.323
AARC-gains	85.438	16.210
AARC-losses	49.261	13.164
Cognitive reserve proxies		
CR-current	1.609	0.335
CR-retrospective	1.192	0.462
Quality of life		
WHOQOL-BREF	67.825	8.958

MMSE, Mini-Mental State Examination; GDS, Geriatric Depression Scale; ATOA, Attitudes Toward Own Aging; AARC, Awareness of Age-Related Change; CR-current, current cognitive reserve; CR-retrospective, retrospective cognitive reserve; WHOQOL, World Health Organization Quality of Life.

### 2.2 Materials

#### 2.2.1 Personal views of aging

*Felt age*. Participants were asked to provide their felt age with a single-item question: “Please indicate the age that you, feel from 0 to 120 years”. Proportional discrepancy scores (dependent variable) were calculated for each participant as a measure of felt age to control for the various effects of chronological age (Debreczeni and Bailey, 2021) as follows: (felt age—chronological age)/chronological age, with negative scores corresponding to feeling younger than one’s own chronological age.

*Attitudes Toward Own Aging scale* (ATOA; Lawton, 1975). It is a 5-item subscale of the Philadelphia Geriatric Center Morale Scale (GCMS; Lawton, 1975) assessing individuals’ overall evaluation of change in their lives as they age. Participants rated their level of agreement with each item on a 4-point Likert scale (from 1 = strongly disagree to 4 = strongly agree). The dependent variable was the sum of the scores for each item, with higher scores corresponding to more positive attitudes towards own aging (max = 20; *α* = 0.710; *ω* = 0.78).

*Awareness of Age-Related Change questionnaire* (AARC; Brothers et al., 2019; Carbone et al., 2024, 2025b). It is a self-report measure designed to assess how much individuals perceive that their lives have changed because of getting older. The AARC comprises 50 items, introduced by a common stem (“With my increasing age, I realize that…”), that assess perceived age-related changes in five key life domains, namely health/physical functioning (e.g., sensory and motor functioning, physical appearance), cognitive functioning (e.g., memory and information processing, knowledge), interpersonal relationships (social support and expectations, sense of belonging), socio-cognitive and socio-emotional functioning (achievement of personal goals, will to live, feelings in response to past, current or expected events), lifestyle and engagement (e.g., autonomy, time and opportunities to plan, organize, and enjoy one’s daily activities); 25 items assess AARC-Gains and 25 items assess AARC-Losses in such different life and behavioural domains (with 5 gain and 5 loss item for each domain). Participants rated how much each item applied to them on a 5-point Likert scale (from 1 = not at all to 5 = very much). The dependent variables were the scores for AARC-Gains and AARC-Losses, calculated by summing the 25 items falling into the respective subscales (max = 125; *α* = 0.917, *ω* = 0.93 and *α* = 0.898, *ω* = 0.93 for AARC-Gains and AARC-Losses, respectively). Higher scores indicate greater AARC-Gains and AARC-Losses, respectively.

#### 2.2.2 Cognitive reserve proxies

*Current and Retrospective Cognitive Reserve survey* (2CR, Borella et al., 2023). The 2CR comprises items spanning five dimensions of experience: socio-economic status (educational level, occupational class, financial wellbeing); leisure activity (e.g., engagement in recreational exercise, creative expression, and intellectual stimulation); social engagement (e.g., participation in volunteering, clubs or public events); spiritual/religious practice (e.g., praying and attending religious rites/ceremonies individually or in group), and family engagement (e.g., partner’s status in terms of physical and mental health, partnership quality, driving and telecommunications usage to keep in contact with relatives). Except for family engagement, these dimensions were assessed with respect to both current status (i.e., late adulthood/older adulthood) -CR-current- and retrospective status (youth or younger adulthood, i.e., ages 20–35/40 years) -CR-retrospective. All response-level items were scaled 0–4, except for educational level, which was scaled from 1 to 7 to cover all of the major educational attainment levels provided by the Italian education and training system (higher scores = higher levels of formal education completed). For the items assessing the engagement in leisure, social, and spiritual/religious activities, participants were asked to rate their frequency of engagement with each of the activities choosing the following options: never, seldom (yearly), sometimes (monthly), often (weekly), always (daily). Two CR-current and CR-retrospective global scores are calculated as the mean of the scores for all their related dimensions: for CR-current, socio-economic status (occupational class, financial wellbeing); family engagement (partner’s status in terms of physical and mental health, partnership quality and connectivity logistics); leisure activity (recreational exercise, creative expression, and intellectual stimulation); social engagement (participation in volunteering, clubs or public events); spiritual/religious practice (praying and attending religious rites/ceremonies individually or in group); for CR-retrospective, socio-economic status (educational level); leisure activity (recreational exercise, creative expression, and participation to public events); social engagement (participation in volunteering and clubs, connectivity logistics); spiritual/religious practice (praying and attending religious rites/ceremonies individually or in group).^1^ Higher scores corresponding to greater CR (see Borella et al., 2023).

#### 2.2.3 Quality of life

*World Health Organization Quality of Life-Bref* (WHOQOL-BREF; Skevington et al., 2004). It is a 26-item questionnaire evaluating QoL, with two questions addressing overall QoL in the past 2 weeks and subsequent items assessing four domains: physical health (7 items), psychological health (6 items), social relationships (3 items), and environment (8 items). Participants rated their agreement with each item on a 5-point Likert scale (from 1 = not at all to 5 = completely). Raw scores were transformed into a 0 to 100-point scale (WHOQOL Group, 1998), and the dependent variable was the total score (*α* = 0.88), where higher scores indicate better QoL.

#### 2.2.4 Covariates

Chronological age, gender (1 = male, 2 = female) and being retired (1 = yes, 2 = no) were considered in the analyses due to their associations with VoA and QoL (e.g., Sabatini et al., 2023a).

### 2.3 Procedure

All participants attended two in-person individual sessions lasting approximately 60 min each, scheduled 1 week apart. Sessions were conducted by a trained experimenter in a quiet setting, to minimize hearing difficulties and potential interruptions.

The questionnaires used in the study protocol were implemented on the Qualtrics platform. To ensure confidentiality and allow data linkage across the two assessment sessions, the experimenter assigned a unique identification code to each participant, which was used consistently across the Qualtrics platform and all paper-based materials.

In the first session, after obtaining their written informed consent, the experimenter guided participants through the completion of the semi-structured interview and the MMSE (using a paper-and-pencil format). Then, participants individually completed the felt age question and the GDS via the Qualtrics platform using a computer provided by the experimenter. In the second session, participants individually completed the following questionnaires: WHOQOL-BREF, AARC, ATOA and 2CR, always via the Qualtrics platform using a computer provided by the experimenter. During questionnaire completion in both sessions, the experimenter remained available to clarify instructions and provide support if needed.

This study was part of a larger project including other measures beyond the scope of this study and therefore not considered here.

### 2.4 Statistical analyses

First, we conducted Pearson’s correlations between sociodemographic variables (chronological age, gender, education), personal VoA (felt age, ATOA, AARC-Gains, AARC-Losses), CR-current and CR-retrospective, and QoL (see Supplementary Table S1).

Then, to simultaneously explore the associations between personal VoA, current and retrospectively reported CR proxy measures, and QoL, we conducted structural equation modeling (SEM; path analysis).

**Figure 1:**
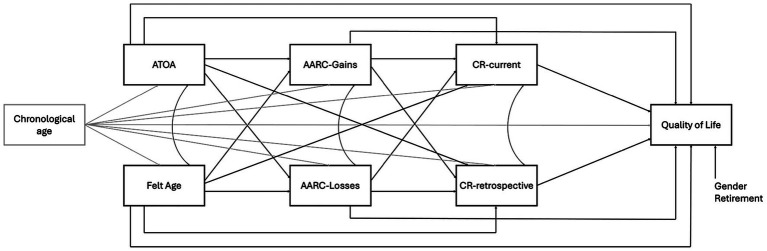
Graphical representation of the estimated model. ATOA, Attitudes Toward Own Aging; AARC, Awareness of Age-related Change; CR-current, current cognitive reserve; CR-retrospective, retrospective cognitive reserve.

More specifically, in line with our hypotheses, we tested a model estimating the following relationships (see Figure 1 for a graphical representation):

(i) Direct effects of felt age, ATOA and AARC on QoL (e.g., Velaithan et al., 2024; Sella et al., 2025a);
(ii) Direct effects of felt age and ATOA on AARC (Gains and Losses) and indirect effects of felt age and ATOA on QoL through AARC (Gains and Losses), since felt age and ATOA captures global evaluations of one’s own aging process likely to prime behaviour-specific, aging-related daily-life experiences depicted by AARC and, in turn, impact health outcomes closely related to QoL (Brothers et al., 2017; Carbone et al., 2025a);
(iii) Direct effects of personal VoA (felt age, ATOA, AARC) on CR-current and CR-retrospective proxy measures (e.g., Beyer et al., 2015; Bu et al., 2024; Robertson and Kenny, 2016; Shoushtari-Moghaddam et al., 2022; Wienert et al., 2017);
(iv) Direct effects of CR-current and CR-retrospective proxy measures on QoL (e.g., Gattuso et al., 2024; Lara et al., 2017; Marzo et al., 2023);
(v) Indirect effects from the different personal VoA dimensions to QoL through CR-current and CR-retrospective proxy measures, to ascertain the mediating role of CR in the VoA-QoL association and, thus, the presence of a behavioural pathway linking personal VoA to QoL (Sabatini et al., 2025; Westerhof et al., 2023; Kim, 2009; Beyer et al., 2015; Li et al., 2013).

Covariances between felt age and ATOA, AARC-Gains and AARC-Losses as well as CR-current and CR-retrospective were also estimated.

Additionally, the direct effect of chronological age on QoL as well as any of its indirect effects on QoL through personal VoA dimensions, CR-current and CR-retrospective were also tested.^2^

Other sociodemographic variables (gender, retirement) were included as covariates, given their influence on QoL.

All parameters were standardized to facilitate interpretation of the results. A well-fitting SEM was defined by the following criteria: Comparative Fit Index (CFI) > 0.95, Standardized Root Mean Square Residual (SRMR) < 0.05, and Root Mean Square Error of Approximation (RMSEA) < 0.08 (Jöreskog and Sörbom, 1993). All analyses were conducted in R using the *lavaan* package.

## 3 Results

The model tested (see Figure 1) showed good fit indices (CFI: 0.993; SRMR: 0.024; RMSEA: 0.044). The predictors together explained 47.91% of the variance in QoL.

**Figure 2:**
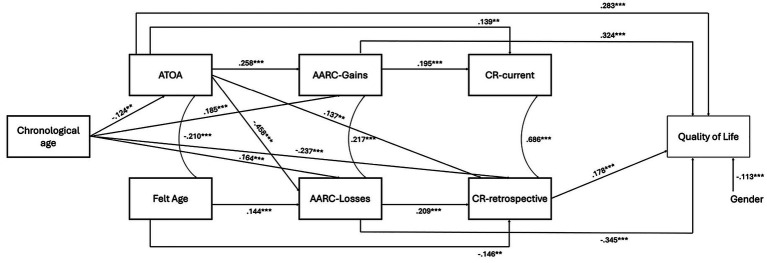
Significant direct effects (standardized solutions) of the model tested. *p* < 0.01; *p* < 0.001. ATOA, Attitudes Toward own Aging; AARC, Awareness of Age-related Change; CR-current, current cognitive reserve; CR-retrospective, retrospective cognitive reserve.

A youthful felt age was related with positive ATOA. Positive ATOA influenced greater AARC-Gains and lower AARC-Losses. In contrast, an older felt age was associated with greater AARC-Losses (see Figure 2). A modest, significant positive association emerged between AARC-Gains and AARC-Losses (see Figure 2).

Positive ATOA and greater AARC-Gains, along with lower AARC-Losses (but not felt age) had a direct association with better QoL (see Figure 2). ATOA was also linked to better QoL through greater AARC-Gains (*β* = 0.084, *p* < 0.001), whereas negative ATOA was associated with poorer QoL through greater AARC-Losses (*β* = 0.158, *p* < 0.001). An older felt age was then associated with poorer QoL through greater AARC-Losses (*β* = −0.050, *p* < 0.001).

Significant total effects of all the personal VoA dimensions emerged, with a youthful felt age (*β* = −0.064, *p* = 0.029), positive ATOA (*β* = 0.603, *p* < 0.001), greater AARC-Gains (*β* = 0.361, *p* < 0.001) and lower AARC-Losses (*β* = −0.361, *p* < 0.001) explaining better QoL.

Positive ATOA and greater AARC-Gains resulted to be associated with higher CR-current, whereas positive ATOA, greater AARC-Losses and a youthful felt age with higher CR-retrospective (see Figure 2).

Then, CR-retrospective, but not CR-current, was found to be directly, positively associated with QoL (see Figure 2).

CR-current also mediated the effect of AARC-Gains on QoL (*β* = 0.035, *p* < 0.001): the greater AARC-Gains, the higher CR-current, the better QoL (see also Supplementary materials). Indirect effects of ATOA through both CR-current (*β* = 0.039, *p* = 0.009) and CR-retrospective (*β* = 0.039, *p* = 0.010) on QoL also emerged: Positive ATOA were associated with higher CR and, in turn, better QoL. No significant indirect effects linking felt age or AARC-Losses to QoL through CR emerged (see Supplementary Table S2).

Direct effects of chronological age emerged only for ATOA (*β* = −0.124, *p* = 0.003), AARC-Gains (*β* = 0.185, *p* < 0.001), AARC-Losses (*β* = 0.164, *p* < 0.001), and CR-retrospective (β = −0.237, *p* < 0.001), with an older chronological age being associated with more negative ATOA, greater AARC-Gains and AARC-Losses and lower CR-retrospective (see Figure 2).

Indirect effects of chronological age on QoL through some of the personal VoA dimensions, but not through CR, emerged (see Supplementary Table S2). An older chronological age was associated with greater AARC-Gains and, in turn, better QoL (*β* = 0.060, *p* < 0.001), while an older chronological age was linked to greater AARC-Losses (*β* = −0.056, *p* < 0.001) and more negative ATOA (*β* = −0.035, *p* < 0.001) and, in turn, to a poorer QoL.

Finally, being female was associated with poorer QoL (see Figure 2), whereas retirement did not show significant associations with QoL (see Supplementary Table S2).

## 4 Discussion

The present cross-sectional study further explored the relationship between various global *vs* behaviour-specific personal VoA dimensions and QoL in community-dwelling middle-aged and older adults. For the first time, at least to our knowledge, it also examined the role CR, in terms of both individual’s retrospective *vs* current stimulating socio-behavioural experiences proxies, in mediating the VoA-QoL associations.

As expected, and in line with previous reports (e.g., Brothers et al., 2017; Carbone et al., 2025a), small-to-medium associations between the global and behaviour-specific personal VoA dimensions considered here emerged: Positive ATOA were associated with greater AARC-Gains, lower AARC-Losses and a youthful felt age, whereas an older felt age was related to greater AARC-Losses only. A significant positive association also emerged between AARC-Gains and AARC-Losses. These findings confirm such personal VoA dimensions being associated yet reflecting different constructs (Brothers et al., 2017, 2019; Sabatini et al., 2021).

Moreover, in line with our hypotheses and previous evidence (e.g., Velaithan et al., 2024; Sella et al., 2025a), nuanced associations emerged between the personal VoA dimensions considered and QoL. Positive ATOA and greater AARC-Gains, along with lower AARC-Losses, were directly associated with better QoL. These findings further confirm that positive global and behaviour-specific personal VoA likely foster a view that focuses on positive aspects of life, whereby contributing to support perceived overall QoL (Velaithan et al., 2024; Sella et al., 2025a). Felt age did not have a direct association with QoL, as expected (Sella et al., 2025a; Carbone et al., 2025a). Such a result could lie in that felt age is operationalized with a single overarching question capturing a comparatively broader self-perception of one’s own aging than the other personal VoA dimensions considered here. These latter capture valenced (both ATOA and AARC) or specific experiences of aging-related changes spanning different, broader everyday life domains of functioning (AARC), more likely contributing to explain overall QoL (Westerhof et al., 2023). It is also worth mentioning that felt age is usually found to be linked to health outcomes closely related to QoL among clinical or more vulnerable populations (Sabatini et al., 2025; Westerhof et al., 2023), which we could not investigate here because of our sample characteristics.

In line with our expectations, ATOA had also an indirect effect on QoL through AARC: Positive ATOA were associated with greater AARC-Gains and, in turn, better QoL, whereas negative ATOA were linked to greater AARC-Losses and, in turn, poorer overall QoL. Interestingly, felt age had an indirect effect, through AARC, on overall QoL: An older felt age was associated with poorer QoL through greater AARC-Losses, whereby further suggesting that felt age may influence health outcomes indirectly, for instance through psychological mechanisms, as previously found (e.g., Wurm et al., 2017; Sella et al., 2025a). Taken together, these results align with previous evidence (e.g., Brothers et al., 2017; Carbone et al., 2025a) and confirm the notion that global evaluations of one’s own aging process (felt age and ATOA) likely prime behaviour-specific, aging-related daily-life experiences depicted by AARC, which, in turn, have a nuanced (i.e., positive or negative) impact on a relevant aspect of healthy/successful aging, here extended to overall QoL.

When adding CR proxies to the picture, a complex and interesting interplay between -current *vs* retrospective- CR, personal VoA dimensions, and QoL emerged. The life-stage dependent nature of CR proxies examined by means of the 2CR survey considered here, in fact, allowed us to capture a differential pattern of associations between CR, personal VoA and QoL, depending on the personal VoA dimensions and life-stage dependent CR proxies considered.

First, it is to note that, consistent with previous evidence (Borella et al., 2023), CR-current and CR-retrospective scores were positively correlated, however they likely capture the CR dynamic nature and reflect distinct set of early life *vs* current enriching socio-behavioural experiences concurring to building CR.

Then, as for personal VoA, positive ATOA were found to be associated with both greater CR-retrospective and CR-current, confirming the role of positive attitudes towards own aging in prompting protective socio-behavioural experiences and habits accumulated across different life stages (retrospective or current). A youthful felt age was also found to be directly associated with greater CR-retrospective, consistently with previous studies showing associations between a younger subjective age and educational attainment (one of the dimensions reflected into the CR-retrospective global score as captured by our 2CR survey; e.g., Ambrosi-Randić et al., 2018; Wettstein et al., 2023), but not CR-current. Such a latter result is, however, in contrast with a few studies showing that a youthful felt age is related to current engagement in social or physical activities (Wienert et al., 2017; Vaartio-Rajalin et al., 2024) or to factors such as occupational attainment (e.g., Barrett, 2003; Ambrosi-Randić et al., 2018; Ye and Post, 2020). The different operationalization of CR proxies (multidimensional -as here- *vs* single proxies) and felt age (which previous studies have often assessed in view of various facets such as mental, physical, and ideal age) might explain these contrasting results and call upon the need of further examining the felt age–CR relationship. As for AARC, greater AARC-Losses were found to be directly associated with greater CR-retrospective, but not CR-current, whereas greater AARC-Gains were associated with greater CR-current, but not CR-retrospective. Previous studies have found that higher educational attainment (a specific proxy of CR-retrospective here) is linked particularly to lower AARC-Losses, but the opposite has also been observed (see Sabatini et al., 2023a). These results are also in line with previous reports (Windsor et al., 2022) showing positive associations between AARC-Gains, but not AARC-Losses, and current engagement in leisure activities (see below for further discussion).

Moreover, CR-retrospective, but not CR-current, was found to be positively associated with overall QoL, in line with our expectations and previous evidence of positive direct associations between CR proxies spanning early-life and later-life socio-behavioural experiences and better overall QoL (Marzo et al., 2023; Lara et al., 2017; Gattuso et al., 2024). Our results, however, disentangle the contribution of current *vs* retrospective/youthful enriching socio-behavioural CR proxies in explaining QoL in midlife and older age, with CR-retrospective playing a major role in prompting QoL. Such a finding can be interpreted in light of the preserved differentiation account, which posits that powerful forms of engagement and enrichment especially early in life contribute to the delineation of endurable individual differences/advantages that persist over the lifespan into older age (Lövdén et al., 2020; Reuter-Lorenz and Park, 2024). Such an account was developed far to explain the “protective role” of CR proxies on cognitive functioning; therefore, its application here remains speculative and warrants expansion through a longitudinal approach. Nonetheless, our results seem to extend this framework also to explain the link between youthful lifestyle CR proxies and overall QoL.

It is noteworthy that CR proxies, as expected, mediated the effects of personal VoA dimensions on QoL, however, and interestingly, depending on the different global *vs* behaviour-specific nature of the personal VoA dimensions as well as the early-life and/or current nature of CR proxies considered.

In particular, indirect effects of positive ATOA explaining better QoL through both higher CR-current and CR-retrospective emerged. ATOA, capturing a global evaluation of one’s aging perception—also rooted in internalized stereotypes across the lifespan into later life (Levy, 2009)—seems to broadly impact, both directly and indirectly, QoL. As such, ATOA have an influence not only on the way in which individuals are aware of gains and losses occurring with aging (AARC-Gains and AARC-Losses), but also on how they proactively draw on resources and enriching experiences accumulated across earlier and current life stages (CR) and, in turn, on their QoL. On the other hand, it is a greater awareness of gain-oriented behaviour-specific, aging-related daily-life experiences, as captured by AARC-Gains, to solicit current resources, strengths, and opportunities, as well as a proactive engagement in enriching behaviours that build up CR, in turn leading to better QoL. Thus, AARC-Gains likely represents a relevant self-regulatory psychological resource helping individuals to proactively engage with the environment and enriching socio-behavioural experiences to adapt to stressors and challenges that increasing age poses, whereby prompting better developmental outcomes like QoL (Windsor et al., 2022; Diehl et al., 2014). Taken together, according to the extant VoA theoretical frameworks (Sabatini et al., 2025; Shrira et al., 2022), previous findings (Kim, 2009; Beyer et al., 2015; Li et al., 2013) and our expectations, our results further suggest that CR resources, that are built through the engagement in diverse socio-behavioural experiences at different life points, may function as a behavioural pathway through which some personal VoA dimensions are likely to be linked to overall QoL.

In contrast, for felt age—the other global personal VoA dimension considered here—no indirect effects through CR proxies on QoL emerged. One possible interpretation could be that feeling younger than ones’ chronological age, which is seen as psychologically distancing oneself from normative aging expectations on one’s “true” age and age peers based on personal aging experiences (Diehl et al., 2014; Wurm et al., 2017), likely impact QoL through psychological mechanisms (AARC-Losses) more than behavioural ones (CR proxies) (Wurm et al., 2017; see also Sella et al., 2025a). Further studies are however needed, possibly considering different nuances of felt age (e.g., mental and physical felt age, ideal age), which was not done here, to clarify these results. Also, although AARC-Losses was associated with CR-retrospective, no indirect effects of AARC-Losses on QoL through CR proxies emerged, whereby suggesting that the relevance of loss-oriented aging-related daily-life experiences characteristics of greater AARC-Losses lead individuals to rely particularly on accumulated CR resources and experiences to appraise aging-related challenges, but other mechanisms likely link it to QoL (Wurm et al., 2017).

Of interest, in line with our expectations and previous findings, chronological age was not linked *per se* to overall QoL (see Sella et al., 2025a). An older chronological age was found to be directly associated with more negative ATOA, as well as with both greater AARC-Gains and AARC-Losses. These results further confirm a coexistence of positive/gain-oriented and negative/loss-oriented evaluations of one’s own aging over the adult life course, which is consistent with previous evidence. ATOA is indeed known to become less positive with advancing age, likely reflecting age-related stereotypes internalization and increased physical and social losses (Lawton, 1975; Miche et al., 2014; Brothers et al., 2017), and some studies have documented positive associations of chronological age with both AARC dimensions, whereby advancing age increase the salience of age-related challenges while also strengthening the recognition of gain-oriented aging-related experiences (Rupprecht et al., 2022; Kaspar et al., 2023; Wettstein et al., 2022). An older chronological age was also found to be associated with lower CR-retrospective, in line with previous reports (see Borella et al., 2023). The indirect effects of chronological age on QoL through some of the personal VoA dimensions, but not through CR, also emerged. An older chronological age was associated with greater AARC-Gains and, in turn, better QoL. In addition, an older chronological age was linked to greater AARC-Losses and more negative ATOA and, in turn, poorer QoL. Such a pattern of findings further underscores how positive and negative personal VoA, linked to aging-related experiences and challenges accumulated over the lifespan into older age, likely impact—in a positive and negative fashion, respectively—relevant outcomes of successful/healthy aging like QoL (Velaithan et al., 2024).

Despite these interesting findings, some limitations of the present study should be acknowledged. First, this was a cross-sectional study spanning the second half of life. Therefore, our study prevents clear conclusions on the causal or bidirectional relationships among VoA, CR proxies, and QoL, which deserves further investigation within a longitudinal and more comprehensive adult lifespan perspective. In this regard, future research is also needed to more clearly disentangle which psychological and behavioural mechanisms and pathways are most relevant for explaining the VoA-QoL associations across different life stages. The model tested here showed a better fit in the full sample, nonetheless additional subgroup analyses (see Supplementary Materials) pointed to some age-specific variations in specific paths, suggesting that the VoA-QoL associations may be primarily explained by psychological mechanisms (AARC) in older age and by behavioural pathways (CR proxies) in middle adulthood. This pattern of findings warrants further investigation (see Kornadt et al., 2020; Wurm et al., 2017). Moreover, the study involved a sample of healthy, well-educated, autonomous individuals, which may limit the generalizability of results to more heterogeneous samples in terms of sociodemographic characteristics and health status. Future studies should also consider other VoA concepts capturing more general, stereotypical beliefs related to the aging process, to better elucidate the link between VoA, CR proxies, and QoL. Also, considering contextual (e.g., sociocultural context, living conditions, life events and role transitions, social connection) and personal (e.g., personality dispositions, life goals, self-regulatory processes) antecedents not included here (e.g., Carbone et al., 2024; Shrira et al., 2022; Sabatini et al., 2025) could be relevant for controlling a wider array of potential confounding factors. Finally, concerning CR, although we rely on a comprehensive set of commonly employed proxies of CR, we lack more objective and multidisciplinary CR assessment methods (e.g., neuroimaging or neurophysiological data). Nonetheless, our study is among the first to examine the associations between the CR concept and a psychological outcome—other than the mostly addressed cognitive functioning—namely, QoL. To our knowledge, it is also the first to provide a comprehensive assessment of the links between personal VoA dimensions, QoL, and life-stage dependent CR proxies.

## 5 Conclusion

To conclude, our results revealed a nuanced pattern of associations between the global *vs* behaviour-specific personal VoA dimensions considered here and overall QoL among middle-aged and older adults. They also suggest that early-life (retrospective) and later-life (current) CR proxies play distinct roles in explaining QoL. For the first time in a comprehensive way, they highlight how well-known proxies contributing to CR’s build up across different life stages likely represent a behavioural pathway through which some specific personal VoA operate to prompt QoL. The differential associations that CR-current and CR-retrospective displayed here with both VoA and QoL support the need to refrain from conceptualizing CR and VoA as static phenomenon/constructs.

Apart from calling for further longitudinal evidence on the links between VoA, life-stage-dependent CR proxies, and QoL, our cross-sectional findings also have important practical implications. They suggest that considering VoA together with modifiable lifestyle (protective- CR proxies) factors may contribute to promoting QoL in later adulthood. Interventions and policies aimed at providing accurate information regarding typical/atypical aging experiences and fostering a growth/positive mental representation and experiences of one’s own aging process, while also encouraging the engagement in enriching lifestyle habits, could represent valuable strategies to support healthy and high-quality aging across adulthood into older age.

## Data Availability

The raw data supporting the conclusions of this article will be made available by the authors, without undue reservation.

## Footnotes

1 There are item-factor structural asymmetries across current vs. retrospective CR dimensions further highlighting the life-stage dependent nature of CR (e.g., connectivity logistics refers to the family engagement dimension for CR-current and to the social engagement dimension for CR-retrospective; participation in public events refers to the social engagement dimension for CR-current, and to the leisure activity dimension for CR-retrospective, and this latter dimension, unlike the CR-current one, does not entail intellectual stimulation activities; see Borella et al., 2023).

2 To further explore age-related differences in the relationships among personal VoA, CR proxies and QoL, additional subgroup analyses were conducted by estimating the same model tested in the full sample separately for middle-aged (50–64 years) and older adults (65–85 years). The model for the older adult group showed acceptable fit indices, whereas the fit indices of the model of the middle-age group likely reflect overfitting. Accordingly, the model estimated on the full sample demonstrated better and more stable/reliable overall fit. Overall, the pattern of associations was consistent across the full sample and the two age groups, although subgroup analyses revealed some age-specific differences in certain direct and indirect paths. These additional analyses are reported in Supplemental Materials (see Tables S3-S7).
