# Supplementary material for: Personal views of aging and quality of life in midlife and older age: the role of cognitive reserve
Source: Front Psychol. 2026 Mar 18;17:1778263. doi: 10.3389/fpsyg.2026.1778263 (PMC13038596; doi:10.3389/fpsyg.2026.1778263)
Supplement: Supplementary file 1 [file Table_1.docx]

**Supplemental Materials**

***Table S1.*** Matrix of correlations among the measures of interest.

|  | Chronological age | 1 | 2 | 3 | 4 | 5 | 6 | 7 | 8 |
| --- | --- | --- | --- | --- | --- | --- | --- | --- | --- |
| Gender (1) | -0.025 |  |  |  |  |  |  |  |  |
| Retirement (2) | -0.665^***^ | 0.160^***^ |  |  |  |  |  |  |  |
| Felt age (3) | -0.042 | -0.032 | 0.058 |  |  |  |  |  |  |
| ATOA (4) | -0.124^**^ | -0.065 | 0.058 | -0.203^***^ |  |  |  |  |  |
| AARC-Gains (5) | 0.151^***^ | 0.037 | -0.155^***^ | -0.005 | 0.224^***^ |  |  |  |  |
| AARC-Losses (6) | 0.215^***^ | 0.074 | -0.124^**^ | 0.231^***^ | -0.507^***^ | 0.095^*^ |  |  |  |
| CR-current (7) | -0.033 | 0.000 | -0.056 | -0.088^*^ | 0.210^***^ | 0.218^***^ | -0.094^*^ |  |  |
| CR-retrospective (8) | -0.209^***^ | 0.067 | 0.147^***^ | -0.115^**^ | 0.080 | -0.027 | 0.051 | 0.649^***^ |  |
| WHOQOL-BREF (9) | -0.010 | -0.150^***^ | -0.063 | -0.186^***^ | 0.572^***^ | 0.396^***^ | -0.484^***^ | 0.307^***^ | 0.045 |

*p<.05, **p<.01, ***p<.001

Notes. ATOA: Attitudes Towards own Aging; AARC: Awareness of Age-Related Change; CR-current: current cognitive reserve; CR-retrospective: retrospective cognitive reserve; WHOQOL: World Health Organization Quality of Life.

Significant, small-to-medium correlations (r from -.50 to .09) emerged between the different personal VoA dimensions considered. Feeling younger than one’s chronological age was negatively related to ATOA, whereas an older subjective age was associated with greater AARC-Losses (see Table S1). Higher ATOA scores were associated, in a positive and negative fashion respectively, with greater AARC-Gains and AARC-Losses (see Table S1). CR-current and CR-retrospective were significantly and positively associated between each other (see Table S1).

All the personal VoA dimensions considered showed small-to-medium correlations (r from .54 to -.15) with QoL: a youthful subjective age, higher ATOA as well as greater AARC-Gains and lower AARC-Losses were associated with higher WHOQOL-BREF scores (see Table S1).

Small, positive correlations emerged between chronological age and AARC-Gains and AARC-Losses, whereas chronological age was negatively associated with ATOA and CR-retrospective (see Table 1). Being female was associated with being employed and poorer QoL. Being employed was associated with lower AARC-Gains and AARC-Losses, whereas being retired with greater CR-retrospective (see Table S1).

***Table S2.*** Standardized direct, indirect, and total effects of chronological age, personal VoA (felt age, ATOA, AARC-Gains and -Losses), proxies of cognitive reserve (current and retrospective) on QoL.

|  | **B** | **SE** | ***z*** | ***p*** | **β** |
| --- | --- | --- | --- | --- | --- |
| **Direct effects on ATOA** |  |  |  |  |  |
| **Chronological age** | **-0.015** | **0.005** | **-2.940** | **0.003** | **-0.124** |
| **Direct effects on felt age** |  |  |  |  |  |
| Chronological age | -0.005 | 0.005 | -0.983 | 0.326 | -0.042 |
| **Direct effects on AARC-Gains** |  |  |  |  |  |
| **ATOA** | **0.258** | **0.042** | **6.154** | **0.000** | **0.258** |
| Felt age | 0.055 | 0.042 | 1.316 | 0.188 | 0.055 |
| **Chronological age** | **0.023** | **0.005** | **4.505** | **0.000** | **0.185** |
| **Direct effects on AARC-Losses** |  |  |  |  |  |
| **ATOA** | **-0.458** | **0.037** | **-12.463** | **0.000** | **-0.458** |
| **Felt age** | **0.144** | **0.036** | **3.957** | **0.000** | **0.144** |
| **Chronological age** | **0.020** | **0.004** | **4.555** | **0.000** | **0.164** |
| **Direct effects on CR-current** |  |  |  |  |  |
| **ATOA** | **0.139** | **0.050** | **2.758** | **0.006** | **0.139** |
| Felt age | -0.055 | 0.042 | -1.305 | 0.192 | -0.055 |
| **AARC-Gains** | **0.195** | **0.044** | **4.453** | **0.000** | **0.195** |
| AARC-Losses | -0.020 | 0.050 | -0.404 | 0.686 | -0.020 |
| Chronological age | -0.005 | 0.005 | -1.016 | 0.310 | -0.043 |
| **Direct effects on CR-retrospective** |  |  |  |  |  |
| **ATOA** | **0.137** | **0.050** | **2.731** | **0.006** | **0.137** |
| **Felt age** | **-0.146** | **0.042** | **-3.448** | **0.001** | **-0.146** |
| AARC-Gains | -0.043 | 0.044 | -0.983 | 0.325 | -0.043 |
| **AARC-Losses** | **0.209** | **0.050** | **4.198** | **0.000** | **0.209** |
| **Chronological age** | **-0.029** | **0.005** | **-5.590** | **0.000** | **-0.237** |
| **Direct effects on WHOQOL** |  |  |  |  |  |
| **ATOA** | **0.282** | **0.036** | **7.776** | **0.000** | **0.283** |
| Felt age | -0.040 | 0.031 | -1.308 | 0.191 | -0.040 |
| **AARC-Gains** | **0.323** | **0.033** | **9.891** | **0.000** | **0.324** |
| **AARC-Losses** | **-0.343** | **0.037** | **-9.292** | **0.000** | **-0.345** |
| CR-current | -0.054 | 0.042 | -1.294 | 0.196 | -0.055 |
| **CR-retrospective** | **0.177** | **0.042** | **4.243** | **0.000** | **0.178** |
| Chronological age | 0.004 | 0.005 | 0.805 | 0.421 | 0.033 |
| **Gender** | **-0.235** | **0.062** | **-3.803** | **0.000** | **-0.113** |
| Retirement | -0.022 | 0.080 | -0.270 | 0.787 | -0.011 |
| **Indirect effects on WHOQOL** |  |  |  |  |  |
| **ATOA🡪 AARC-Gains** | **0.083** | **0.016** | **5.225** | **0.000** | **0.084** |
| **ATOA🡪 AARC-Losses** | **0.157** | **0.021** | **7.450** | **0.000** | **0.158** |
| Felt age🡪 AARC-Gains | 0.018 | 0.014 | 1.304 | 0.192 | 0.018 |
| **Felt age🡪 AARC-Losses** | **-0.050** | **0.014** | **-3.641** | **0.000** | **-0.050** |
| **ATOA🡪CR-current** | **0.039** | **0.015** | **2.599** | **0.009** | **0.039** |
| **ATOA🡪CR-retrospective** | **0.039** | **0.015** | **2.577** | **0.010** | **0.039** |
| Felt age🡪CR-current | 0.002 | 0.002 | 0.924 | 0.356 | 0.002 |
| Felt age🡪CR-retrospective | 0.006 | 0.005 | 1.223 | 0.221 | 0.006 |
| **AARC-Gains🡪CR-current** | **0.035** | **0.011** | **3.072** | **0.002** | **0.035** |
| AARC-Gains🡪CR-retrospective | 0.002 | 0.003 | 0.783 | 0.434 | 0.002 |
| AARC-Losses🡪CR-current | -0.004 | 0.009 | -0.402 | 0.688 | -0.004 |
| AARC-Losses🡪CR-retrospective | -0.011 | 0.009 | -1.237 | 0.216 | -0.011 |
| **Chronological age🡪ATOA** | **-0.004** | **0.002** | **-2.750** | **0.006** | **-0.035** |
| Chronological age🡪Felt age | 0.000 | 0.000 | 0.786 | 0.432 | 0.002 |
| **Chronological age🡪AARC-Gains** | **0.007** | **0.002** | **4.100** | **0.000** | **0.060** |
| **Chronological age🡪AARC-Losses** | **-0.007** | **0.002** | **-4.090** | **0.000** | **-0.056** |
| Chronological age🡪CR-current | -0.001 | 0.001 | -0.988 | 0.323 | -0.008 |
| Chronological age🡪CR-retrospective | 0.002 | 0.001 | 1.261 | 0.207 | 0.013 |
| **Total effects** |  |  |  |  |  |
| **ATOA** | **0.600** | **0.050** | **11.971** | **0.000** | **0.603** |
| **Felt age** | **-0.064** | **0.029** | **-2.186** | **0.029** | **-0.064** |
| **AARC-Gains** | **0.360** | **0.032** | **11.284** | **0.000** | **0.361** |
| **AARC-Losses** | **-0.358** | **0.036** | **-9.824** | **0.000** | **-0.360** |
| Chronological age | 0.001 | 0.005 | 0.191 | 0.849 | 0.009 |
| **Covariances** |  |  |  |  |  |
| **ATOA~~felt age** | **-0.208** | **0.043** | **-4.830** | **0.000** | **-0.210** |
| **AARC-Gains~~AARC-Losses** | **0.173** | **0.035** | **4.981** | **0.000** | **0.217** |
| **CR-current~~CR-retrospective** | **0.627** | **0.047** | **13.282** | **0.000** | **0.686** |

| **Variances** | **B** | **SE** | ***z*** | ***p*** | **β** |
| --- | --- | --- | --- | --- | --- |
| ATOA | 0.983 | 0.059 | 16.598 | 0.000 | 0.985 |
| Felt age | 0.996 | 0.060 | 16.598 | 0.000 | 0.998 |
| AARC-Gains | 0.913 | 0.055 | 16.598 | 0.000 | 0.914 |
| AARC-Losses | 0.698 | 0.042 | 16.598 | 0.000 | 0.699 |
| CR-current | 0.919 | 0.055 | 16.598 | 0.000 | 0.920 |
| CR-retrospective | 0.909 | 0.055 | 16.598 | 0.000 | 0.911 |
| WHOQOL | 0.467 | 0.028 | 16.598 | 0.000 | 0.472 |
|  |  |  |  |  |  |

Notes. ATOA: Attitudes Towards own Aging; AARC: Awareness of Age-Related Change; CR-current: current cognitive reserve; CR-retrospective: retrospective cognitive reserve; WHOQOL: World Health Organization Quality of Life.

**Sub-group analyses: middle-aged vs older adults**

To examine potential influences due to life transitions (e.g., retirement or subjective experience of entering older adulthood), we run the same model by splitting the sample into middle-aged (50-64 years old) and older adults (65-84 years old). This age-range choice is justified by the effective average retirement age of 64.8 years, reflecting the use of early retirement schemes and pension practices, as shown by the most recent reports from INPS (Italy’s National Institute for Social Security) and the OECD (Organisation for Economic Cooperation and Development).

Table S3 shows the descriptive statistics of participants socio-demographic characteristics, screening measures and measures of interest by age group.

***Table S3***. Descriptive statistics of participants’ sociodemographic characteristics, screening measures, and measures of interest by age group.

|  | Middle-aged adults  (age range: 50-64; N=325) | | Older adults  (age range: 65-84; N=227) | |
| --- | --- | --- | --- | --- |
|  | *M* | *SD* | *M* | *SD* |
| **Socio-demographic characteristics** |  |  |  |  |
| Chronological age | 58.129 | 4.179 | 71.921 | 5.125 |
| Education (years) | 12.412 | 3.571 | 10.427 | 4.336 |
| Gender, n females (%) | 216 (66%) | - | 137 (60%) |  |
| Retirement (yes, %) | 55 (17%) | - | 187 (82%) |  |
| **Screening measures** |  |  |  |  |
| Mini-Mental State Examination |  |  | 29.35 | 1.01 |
| Geriatric depression scale | 1.289 | 1.320 | 1.176 | 1.298 |
| **Views of aging** |  |  |  |  |
| Felt age | -0.128 | 0.156 | -0.142 | 0.141 |
| ATOA | 12.366 | 1.288 | 12.480 | 1.371 |
| AARC-Gains | 83.597 | 16.148 | 88.075 | 15.966 |
| AARC-Losses | 47.495 | 12.927 | 51.789 | 13.116 |
| **Cognitive reserve proxies** |  |  |  |  |
| CR-current | 1.612 | 0.340 | 1.604 | 0.328 |
| CR-retrospective | 1.269 | 0.446 | 1.083 | 0.462 |
| **Quality of life** |  |  |  |  |
| WHOQOL-BREF | 67.796 | 9.009 | 67.866 | 8.903 |

Notes. ATOA: Attitudes Towards own Aging; AARC: Awareness of Age-Related Change; CR-current: current cognitive reserve; CR-retrospective: retrospective cognitive reserve; WHOQOL: World Health Organization Quality of Life.

The reliability of personal VoA measures with ordinal scales across these two age subgroups showed consistently adequate-to-good/excellent indices (50-64 years: ATOA ω = .79, AARC Gains ω =.93, AARC Losses ω =.93; 65-84 years: ATOA ω =.78, AARC Gains ω =.94, AARC Losses ω =.89).

Results of the models for middle-aged (50-64 years) and older adults (65-84 years) are shown in Table S4 and Table S5, respectively.

***Table S4.*** Standardized direct, indirect, and total effects of chronological age, personal views on aging (felt age, ATOA, AARC-Gains and AARC-Losses), and proxies of cognitive reserve (current and retrospective) on QoL in the middle-aged group (50-64 years).

|  | **B** | **SE** | ***z*** | ***p*** | **β** |
| --- | --- | --- | --- | --- | --- |
| **Direct effects on ATOA** |  |  |  |  |  |
| Chronological age | -0.005 | 0.013 | -0.366 | 0.715 | -0.020 |
| **Direct effects on felt age** |  |  |  |  |  |
| **Chronological age** | **-0.034** | **0.013** | **-2.620** | **0.009** | **-0.144** |
| **Direct effects on AARC-Gains** |  |  |  |  |  |
| **ATOA** | **0.210** | **0.055** | **3.825** | **0.000** | **0.210** |
| Felt age | 0.015 | 0.056 | 0.273 | 0.785 | 0.015 |
| **Chronological age** | **0.039** | **0.013** | **2.997** | **0.003** | **0.162** |
| **Direct effects on AARC-Losses** |  |  |  |  |  |
| **ATOA** | **-0.466** | **0.050** | **-9.398** | **0.000** | **-0.466** |
| Felt age | 0.090 | 0.050 | 1.799 | 0.072 | 0.090 |
| Chronological age | 0.005 | 0.012 | 0.388 | 0.698 | 0.019 |
| **Direct effects on CR-current** |  |  |  |  |  |
| ATOA | 0.123 | 0.064 | 1.916 | 0.055 | 0.123 |
| Felt age | 0.007 | 0.055 | 0.134 | 0.893 | 0.007 |
| **AARC-Gains** | **0.220** | **0.057** | **3.897** | **0.000** | **0.220** |
| AARC-Losses | 0.046 | 0.063 | 0.737 | 0.461 | 0.046 |
| Chronological age | 0.013 | 0.013 | 1.015 | 0.310 | 0.055 |
| **Direct effects on CR-retrospective** |  |  |  |  |  |
| **ATOA** | **0.130** | **0.065** | **2.011** | **0.044** | **0.130** |
| **Felt age** | **-0.116** | **0.056** | **-2.069** | **0.039** | **-0.116** |
| AARC-Gains | -0.052 | 0.057 | -0.912 | 0.362 | -0.052 |
| **AARC-Losses** | **0.284** | **0.063** | **4.489** | **0.000** | **0.284** |
| Chronological age | -0.014 | 0.013 | -1.085 | 0.278 | -0.060 |
| **Direct effects on WHOQOL** |  |  |  |  |  |
| **ATOA** | **0.306** | **0.050** | **6.177** | **0.000** | **0.306** |
| Felt age | -0.024- | 0.043 | -0.555 | 0.579 | 0.024 |
| **AARC-Gains** | **0.259** | **0.046** | **5.633** | **0.000** | **0.259** |
| **AARC-Losses** | **-0.318** | **0.050** | **-6.310** | **0.000** | **-0.318** |
| CR-current | -0.070 | 0.060 | -1.172 | 0.241 | -0.070 |
| **CR-retrospective** | **0.156** | **0.060** | **2.580** | **0.010** | **0.156** |
| Chronological age | 0.009 | 0.011 | 0.848 | 0.397 | 0.039 |
| **Gender** | **-0.231** | **0.088** | **-2.626** | **0.009** | **-0.110** |
| Retirement | -0.110 | 0.121 | -0.910 | 0.363 | -0.041 |
| **Indirect effects on WHO-QoL** |  |  |  |  |  |
| **ATOA🡪 AARC-Gains** | **0.010** | **0.004** | **2.646** | **0.008** | **0.042** |
| ATOA🡪 AARC-Losses | -0.001 | 0.004 | -0.387 | 0.698 | -0.006 |
| Felt age🡪 AARC-Gains | 0.001 | 0.002 | 0.543 | 0.587 | 0.003 |
| Felt age🡪 AARC-Losses | -0.001 | 0.004 | -0.365 | 0.715 | -0.006 |
| ATOA🡪CR-current | 0.038 | 0.021 | 1.830 | 0.067 | 0.038 |
| ATOA🡪CR-retrospective | 0.040 | 0.021 | 1.912 | 0.056 | 0.040 |
| Felt age🡪CR-current | -0.000 | 0.001 | -0.131 | 0.896 | -0.000 |
| Felt age🡪CR-retrospective | 0.003 | 0.005 | 0.536 | 0.592 | 0.003 |
| **AARC-Gains🡪CR-current** | **0.034** | **0.016** | **2.151** | **0.031** | **0.034** |
| AARC-Gains🡪CR-retrospective | 0.004 | 0.005 | 0.720 | 0.472 | 0.004 |
| AARC-Losses🡪CR-current | 0.007 | 0.010 | 0.708 | 0.479 | 0.007 |
| AARC-Losses🡪CR-retrospective | -0.020 | 0.018 | -1.134 | 0.257 | -0.020 |
| Chronological age🡪ATOA | 0.001 | 0.001 | 0.796 | 0.426 | 0.004 |
| Chronological age🡪Felt age | 0.002 | 0.002 | 0.944 | 0.345 | 0.009 |
| Chronological age🡪AARC-Gains | 0.004 | 0.014 | 0.273 | 0.785 | 0.004 |
| Chronological age🡪AARC-Losses | -0.029 | 0.017 | -1.730 | 0.084 | -0.029 |
| **Chronological age🡪CR-current** | **0.055** | **0.017** | **3.164** | **0.002** | **0.055** |
| **Chronological age🡪CR-retrospective** | **0.148** | **0.028** | **5.239** | **0.000** | **0.148** |
| **Total effects** |  |  |  |  |  |
| **ATOA** | **0.384** | **0.072** | **5.324** | **0.000** | **0.384** |
| Felt age | -0.021 | 0.038 | -0.554 | 0.579 | -0.021 |
| **AARC-Gains** | **0.297** | **0.044** | **6.791** | **0.000** | **0.297** |
| **AARC-Losses** | **-0.330** | **0.049** | **-6.804** | **0.000** | **-0.331** |
| Chronological age | 0.020 | 0.012 | 1.645 | 0.100 | 0.085 |
| **Covariances** |  |  |  |  |  |
| **ATOA~~felt age** | **-0.224** | **0.056** | **-3.994** | **0.000** | **-0.227** |
| **AARC-Gains~~AARC-Losses** | **0.189** | **0.048** | **3.972** | **0.000** | **0.226** |
| **CR-current~~CR-retrospective** | **0.657** | **0.063** | **10.450** | **0.000** | **0.711** |

| **Variances** | **B** | **SE** | ***z*** | ***p*** | **β** |
| --- | --- | --- | --- | --- | --- |
| ATOA | 0.997 | 0.078 | 12.748 | 0.000 | 1.000 |
| Felt age | 0.976 | 0.077 | 12.748 | 0.000 | 0.979 |
| AARC-Gains | 0.930 | 0.073 | 12.748 | 0.000 | 0.933 |
| AARC-Losses | 0.754 | 0.059 | 12.748 | 0.000 | 0.756 |
| CR-current | 0.918 | 0.072 | 12.748 | 0.000 | 0.920 |
| CR-retrospective | 0.929 | 0.073 | 12.748 | 0.000 | 0.932 |
| WHOQOL | 0.536 | 0.042 | 12.748 | 0.000 | 0.539 |

Notes. ATOA: Attitudes Towards own Aging; AARC: Awareness of Age-Related Change; CR-current: current cognitive reserve; CR-retrospective: retrospective cognitive reserve; WHOQOL: World Health Organization Quality of Life.

***Table S5.*** Standardized direct, indirect, and total effects of chronological age, personal views on aging (felt age, ATOA, AARC-Gains and AARC-Losses), and proxies of cognitive reserve (current and retrospective) on QoL in the middle-aged group (65-84 years old).

|  | **B** | **SE** | ***z*** | ***p*** | **β** |
| --- | --- | --- | --- | --- | --- |
| **Direct effects on ATOA** |  |  |  |  |  |
| **Chronological age** | **-0.035** | **0.013** | **-2.727** | **0.006** | **-0.179** |
| **Direct effects on felt age** |  |  |  |  |  |
| Chronological age | -0.012 | 0.013 | -0.957 | 0.339 | -0.064 |
| **Direct effects on AARC-Gains** |  |  |  |  |  |
| **ATOA** | **0.313** | **0.065** | **4.790** | **0.000** | **0.313** |
| Felt age | 0.124 | 0.065 | 1.921 | 0.055 | 0.124 |
| Chronological age | 0.006 | 0.013 | 0.461 | 0.645 | 0.030 |
| **Direct effects on AARC-Losses** |  |  |  |  |  |
| **ATOA** | **-0.442** | **0.055** | **-8.067** | **0.000** | **-0.442** |
| **Felt age** | **0.231** | **0.054** | **4.283** | **0.000** | **0.231** |
| **Chronological age** | **0.045** | **0.011** | **4.302** | **0.000** | **0.232** |
| **Direct effects on CR-current** |  |  |  |  |  |
| **ATOA** | **0.175** | **0.078** | **2.261** | **0.024** | **0.175** |
| **Felt age** | **-0.148** | **0.065** | **-2.262** | **0.024** | **-0.148** |
| AARC-Gains | 0.125 | 0.066 | 1.886 | 0.059 | 0.125 |
| AARC-Losses | -0.071 | 0.079 | -0.896 | 0.370 | -0.071 |
| **Chronological age** | **-0.029** | **0.013** | **-2.289** | **0.022** | **-0.149** |
| **Direct effects on CR-retrospective** |  |  |  |  |  |
| ATOA | 0.152 | 0.082 | 1.870 | 0.061 | 0.152 |
| **Felt age** | **-0.180** | **0.069** | **-2.620** | **0.009** | **-0.180** |
| AARC-Gains | -0.039 | 0.070 | -0.558 | 0.577 | -0.039 |
| AARC-Losses | 0.115 | 0.083 | 1.384 | 0.166 | 0.115 |
| Chronological age | -0.026 | 0.013 | -1.949 | 0.051 | -0.134 |
| **Direct effects on WHOQOL** |  |  |  |  |  |
| **ATOA** | **0.218** | **0.058** | **3.776** | **0.000** | **0.220** |
| Felt age | -0.007 | 0.049 | -0.152 | 0.879 | -0.007 |
| **AARC-Gains** | **0.395** | **0.050** | **7.947** | **0.000** | **0.398** |
| **AARC-Losses** | **-0.295** | **0.059** | **-4.984** | **0.000** | **-0.297** |
| CR-current | -0.053 | 0.061 | -0.874 | 0.382 | -0.054 |
| **CR-retrospective** | **0.214** | **0.064** | **3.334** | **0.001** | **0.216** |
| Chronological age | 0.006 | 0.010 | 0.584 | 0.559 | 0.029 |
| **Gender** | **-0.256** | **0.093** | **-2.748** | **0.006** | **-0.127** |
| Retirement | 0.079 | 0.122 | 0.646 | 0.518 | 0.030 |
| **Indirect effects on WHO-QoL** |  |  |  |  |  |
| **ATOA🡪 AARC-Gains** | **0.124** | **0.030** | **4.103** | **0.000** | **0.125** |
| **ATOA🡪 AARC-Losses** | **0.130** | **0.031** | **4.240** | **0.000** | **0.131** |
| Felt age🡪 AARC-Gains | 0.049 | 0.026 | 1.868 | 0.062 | 0.049 |
| **Felt age🡪 AARC-Losses** | **-0.068** | **0.021** | **-3.248** | **0.001** | **-0.069** |
| ATOA🡪CR-current | 0.038 | 0.020 | 1.940 | 0.052 | 0.039 |
| ATOA🡪CR-retrospective | 0.033 | 0.020 | 1.676 | 0.094 | 0.033 |
| Felt age🡪CR-current | 0.001 | 0.007 | 0.151 | 0.880 | 0.001 |
| Felt age🡪CR-retrospective | 0.001 | 0.009 | 0.151 | 0.880 | 0.001 |
| AARC-Gains🡪CR-current | 0.027 | 0.016 | 1.642 | 0.101 | 0.027 |
| AARC-Gains🡪CR-retrospective | 0.002 | 0.004 | 0.470 | 0.638 | 0.002 |
| AARC-Losses🡪CR-current | -0.015 | 0.018 | -0.865 | 0.387 | -0.015 |
| AARC-Losses🡪CR-retrospective | -0.006 | 0.008 | -0.739 | 0.460 | -0.006 |
| **Chronological age🡪ATOA** | **-0.008** | **0.003** | **-2.211** | **0.027** | **-0.039** |
| Chronological age🡪Felt age | 0.000 | 0.001 | 0.150 | 0.881 | 0.000 |
| Chronological age🡪AARC-Gains | 0.002 | 0.005 | 0.461 | 0.645 | 0.012 |
| **Chronological age🡪AARC-Losses** | **-0.013** | **0.004** | **-3.257** | **0.001** | **-0.069** |
| Chronological age🡪CR-current | -0.006 | 0.003 | -1.887 | 0.059 | -0.032 |
| Chronological age🡪CR-retrospective | 0.001 | 0.002 | 0.797 | 0.425 | 0.007 |
| **Total effects** |  |  |  |  |  |
| **ATOA** | **0.289** | **0.083** | **3.492** | **0.000** | **0.292** |
| Felt age | -0.005 | 0.033 | -0.152 | 0.879 | -0.005 |
| **AARC-Gains** | **0.424** | **0.050** | **8.440** | **0.000** | **0.427** |
| **AARC-Losses** | **-0.316** | **0.060** | **-5.273** | **0.000** | **-0.319** |
| Chronological age | -0.018 | 0.011 | -1.605 | 0.108 | -0.092 |
| **Covariances** |  |  |  |  |  |
| **ATOA~~felt age** | **-0.186** | **0.066** | **-2.810** | **0.005** | **-0.190** |
| **AARC-Gains~~AARC-Losses** | **0.166** | **0.051** | **3.249** | **0.001** | **0.221** |
| **CR-current~~CR-retrospective** | **0.579** | **0.071** | **8.191** | **0.000** | **0.650** |

| **Variances** | **B** | **SE** | ***z*** | ***p*** | **β** |
| --- | --- | --- | --- | --- | --- |
| ATOA | 0.964 | 0.091 | 10.630 | 0.000 | 0.968 |
| Felt age | 0.992 | 0.093 | 10.630 | 0.000 | 0.996 |
| AARC-Gains | 0.899 | 0.085 | 10.630 | 0.000 | 0.903 |
| AARC-Losses | 0.629 | 0.059 | 10.630 | 0.000 | 0.632 |
| CR-current | 0.849 | 0.080 | 10.630 | 0.000 | 0.853 |
| CR-retrospective | 0.937 | 0.088 | 10.630 | 0.000 | 0.941 |
| WHOQOL | 0.459 | 0.043 | 10.630 | 0.000 | 0.468 |

*Note:* ATOA: Attitudes Towards own Aging; AARC: Awareness of Age-Related Change; CR-current: current cognitive reserve; CR-retrospective: retrospective cognitive reserve; WHOQOL: World Health Organization Quality of Life.

A comparison of model fit indices between the full sample and the two age groups is presented in Table S6, while Table S7 provides a summary of parameter significance for the full-sample model and the age-group-specific models.

The model for the older adult group showed acceptable fit indices, whereas the fit indices of the model of the middle-age group likely reflect overfitting; the model estimated on the full sample demonstrated better and more stable/reliable overall fit indices.

***Table S6.*** Comparison of model fit indices between the full sample and age-group-specific SEM models.

| **Age group** | **χ²** | **df** | **p-value** | **RMSEA** | **SRMR** | **CFI** | **NNFI** |
| --- | --- | --- | --- | --- | --- | --- | --- |
| 50-64 years | 9.717 | 12 | 0.641 | 0.000 | 0.024 | 1.000 | 1.000 |
| 65-84 years | 24.444 | 12 | 0.018 | 0.068 | 0.046 | 0.984 | 0.944 |
| Full: 50-84 years | 24.700 | 12 | 0.016 | 0.044 | 0.024 | 0.992 | 0.973 |

Overall, the pattern of associations was consistent across the full sample and the two age groups, with some differences in specific paths.

An older felt age was associated with greater AARC-Losses in the older adult group (β = 0.231, p < .001), but not in the middle-aged one. Both ATOA and felt age were indirectly associated with overall QoL through AARC-Losses only among the older adult group (β = 0.131, p < .001 and β = -0.069, p < .001, respectively), but not in the middle-aged one.

ATOA showed a direct, positive association with CR-current in the older adult group (β = 0.175, p = .024) but not in middle-aged individuals, whereas a direct, positive association between ATOA and CR-retrospective emerged in the middle-aged group (β = 0.130, p = .044), but not in the older adult one. Moreover, a youthful felt age emerged to be directly associated with CR-current in the older adult group (β = -0.148, p = .024) but not in the middle-aged one.

AARC-Gains were positively associated with CR-current in the middle-aged group (β = 0.220, p < .001) but not in the older adult group, while AARC-Losses were positively associated with CR-retrospective in the middle-aged group (β = 0.284, p < .001) but not in the older adults group. The indirect effects of ATOA on overall QoL through both CR-current and CR-retrospective were non-significant in both age groups. The indirect effect of AARC-Gains on overall QoL through CR-current was significant among middle-aged individuals (β = 0.034, p = .031), but not in the older adult group.

Among the older adult group, but not in the middle-aged group, chronological age was associated with ATOA (β = -0.179, p = .006), AARC-Losses (β = 0.232, p < .001) and CR-current (β = -0.149, p = .022). In the middle-aged group, but not among older adults, chronological age was associated with AARC-Gains (β = 0.162, p = .003) and felt age (β = -0.144, p = .009). The direct effect of chronological age on CR-retrospective was non-significant in both age groups. Then, the indirect effects of chronological age on overall QoL through both ATOA and AARC-Losses were significant among older adults (β = -0.039, p = .027 and β = -0.069, p = .001, respectively), but not in the middle-aged group. The indirect effect of chronological age on overall QoL through AARC-Gains was non-significant in both age groups. Indirect effects of chronological age on overall QoL through both CR-current and CR-retrospective emerged among middle-aged individuals (β = 0.055, p = .002 and β = 0.148, p < .001, respectively), but not in the older adult group.

All other significant paths identified in the full-sample model were confirmed in both age groups.

***Table S7.*** Summary of parameter significance by total sample and across age groups, indicating which paths are significant (✓) or non-significant (NS).

|  | **Total sample**  **(50-84 years)** | **Middle-aged group**  **(50-64 years)** | **Older adult group**  **(65-84 years)** |
| --- | --- | --- | --- |
| **Direct effects on ATOA** |  |  |  |
| Chronological age | ✓ | **NS** | ✓ |
| **Direct effects on felt age** |  |  |  |
| Chronological age | NS | **✓** | NS |
| **Direct effects on AARC-Gains** |  |  |  |
| ATOA | ✓ | ✓ | ✓ |
| Felt age | NS | NS | NS |
| Chronological age | ✓ | ✓ | **NS** |
| **Direct effects on AARC-Losses** |  |  |  |
| ATOA | ✓ | ✓ | ✓ |
| Felt age | ✓ | **NS** | ✓ |
| Chronological age | ✓ | **NS** | ✓ |
| **Direct effects on CR-current** |  |  |  |
| ATOA | ✓ | **NS** | ✓ |
| Felt age | NS | NS | **✓** |
| AARC-Gains | ✓ | ✓ | **NS** |
| AARC-Losses | NS | NS | NS |
| Chronological age | NS | NS | **✓** |
| **Direct effects on CR-retrospective** |  |  |  |
| ATOA | ✓ | ✓ | **NS** |
| Felt age | ✓ | ✓ | ✓ |
| AARC-Gains | NS | NS | NS |
| AARC-Losses | ✓ | ✓ | **NS** |
| Chronological age | ✓ | **NS** | **NS** |
| **Direct effects on WHOQOL** |  |  |  |
| ATOA | ✓ | ✓ | ✓ |
| Felt age | NS | NS | NS |
| AARC-Gains | ✓ | ✓ | ✓ |
| AARC-Losses | ✓ | ✓ | ✓ |
| CR-current | NS | NS | NS |
| CR-retrospective | ✓ | ✓ | ✓ |
| Chronological age | NS | NS | NS |
| Gender | ✓ | ✓ | ✓ |
| Retirement | NS | NS | NS |
| **Indirect effects on WHOQOL** |  |  |  |
| ATOA🡪 AARC-Gains | ✓ | ✓ | ✓ |
| ATOA🡪 AARC-Losses | ✓ | **NS** | ✓ |
| Felt age🡪 AARC-Gains | NS | NS | NS |
| Felt age🡪 AARC-Losses | ✓ | **NS** | ✓ |
| ATOA🡪CR-current | ✓ | **NS** | **NS** |
| ATOA🡪CR-retrospective | ✓ | **NS** | **NS** |
| Felt age🡪CR-current | NS | NS | NS |
| Felt age🡪CR-retrospective | NS | NS | NS |
| AARC-Gains🡪CR-current | ✓ | ✓ | **NS** |
| AARC-Gains🡪CR-retrospective | NS | NS | NS |
| AARC-Losses🡪CR-current | NS | NS | NS |
| AARC-Losses🡪CR-retrospective | NS | NS | NS |
| Chronological age🡪ATOA | ✓ | **NS** | ✓ |
| Chronological age🡪Felt age | NS | NS | NS |
| Chronological age🡪AARC-Gains | ✓ | **NS** | **NS** |
| Chronological age🡪AARC-Losses | ✓ | **NS** | ✓ |
| Chronological age🡪CR-current | NS | **✓** | NS |
| Chronological age🡪CR-retrospective | NS | **✓** | NS |
| **Total effects** |  |  |  |
| ATOA | ✓ | ✓ | ✓ |
| Felt age | ✓ | **NS** | **NS** |
| AARC-Gains | ✓ | ✓ | ✓ |
| AARC-Losses | ✓ | ✓ | ✓ |
| Chronological age | NS | NS | NS |
| **Covariances** |  |  |  |
| ATOA~~felt age | ✓ | ✓ | ✓ |
| AARC-Gains~~AARC-Losses | ✓ | ✓ | ✓ |
| CR-current~~CR-retrospective | ✓ | ✓ | ✓ |
